# Long-term subjective and objective outcomes after digital nerve repair: a cohort study

**DOI:** 10.1177/17531934241286116

**Published:** 2024-10-13

**Authors:** Linda Evertsson, Anders Björkman, Christina Turesson, Marianne Arner, Cecilia Mellstrand Navarro

**Affiliations:** 1 Department of Clinical Science and Education, Karolinska Institutet, Department of Hand Surgery, Södersjukhuset Hospital, Stockholm, Sweden; 2 Department of Hand Surgery, Institute of Clinical Sciences, Sahlgrenska University Hospital and Sahlgrenska Academy, University of Gothenburg, Sweden; 3 Department of Health, Medicine and Caring Sciences, Division of Prevention, Rehabilitation and Community Medicine, Linkoping University, Norrkoping, Sweden; 4 Department of Clinical Sciences Danderyd Hospital, Section for Orthopaedics, Stockholm, Sweden

**Keywords:** Digital nerve injury, nerve suture, sensory function, hand function, hand injury, peripheral nerve injury

## Abstract

Digital nerve injuries are common, but few studies report long-term effects for the individual. The primary aim of this matched-pairs study comparing digital nerve injuries in border digits or central fingers was to investigate hand function 3–10 years after digital nerve repair, assessed using the Mini Sollerman test in 86 patients. Secondary outcomes were sensory function, range of motion, grip strength and patient-reported measures. No significant difference was seen in hand function between the groups, except for lower grip strength in patients with central finger injury. Tactile discrimination was achieved in 87%, with best results among participants aged less than 44 years. Touch perception was measurable in 99%. No statistically significant differences in sensory function were found between the groups. Patient-reported disability was low, with median Quick Disabilities of the Arm, Shoulder and Hand score of 5, but half of the patients reported neuropathic pain. Numbness and cold sensitivity were the symptoms graded worst after digital nerve injury.

**Level of evidence:** III

## Introduction

Digital nerve injuries occur frequently. However, the extent to which a digital nerve injury affects hand function and perception of health is unknown. Moreover, few studies address rehabilitation after digital nerve injuries and consensus is lacking both regarding the optimal rehabilitation and how to assess outcome. A digital nerve injury can cause impaired discriminative sensation, pain, numbness and cold sensitivity in the fingers (Frostadottir et al., 2022; Thorsén et al., 2012), all of which can negatively affect hand function (Lundborg, 2004). To our knowledge, there are no previous studies describing long-term results and function such as return of sensation, pain and fine motor function in combination with patient-reported outcome measures (PROMs) after digital nerve injuries. In addition, there are no previous studies addressing if outcome after a digital nerve injury differs depending on which finger was injured (Dunlop et al., 2019). Hypothetically, digital nerve injuries to border digits (thumb, index and little finger) could lead to worse function after injury, since border digits are important for hand function. Conversely, as these skin areas are more exposed to sensory stimuli (Sollerman, 1980), this could potentially prompt more active rehabilitation through daily activities with superior hand function and sensory outcome.

The primary aim of this matched-pair cohort study was to investigate if hand function assessed with the Mini Sollerman test was more impaired after digital nerve injury in the thumb, index or little finger, compared with injury to the central fingers, 3–10 years after digital nerve injury and repair. Secondary aims were assessment of sensory function, range of motion, grip strength, neuropathic pain, anxiety and depression, and physical activity levels.

## Methods

### Participants

This matched-pairs cohort study included patients treated surgically for a single digital nerve injury between 2012 and 2019 at the Department of Hand Surgery, Södersjukhuset Hospital in Stockholm, a second-level trauma centre providing specialized hand surgery care for 2.5 million citizens (Statistics CoS, 2023). This study received ethical approval and was conducted according to the Declaration of Helsinki (Williams, 2008). All participants received oral and written information on the study and signed a letter of consent.

**Figure 1.**
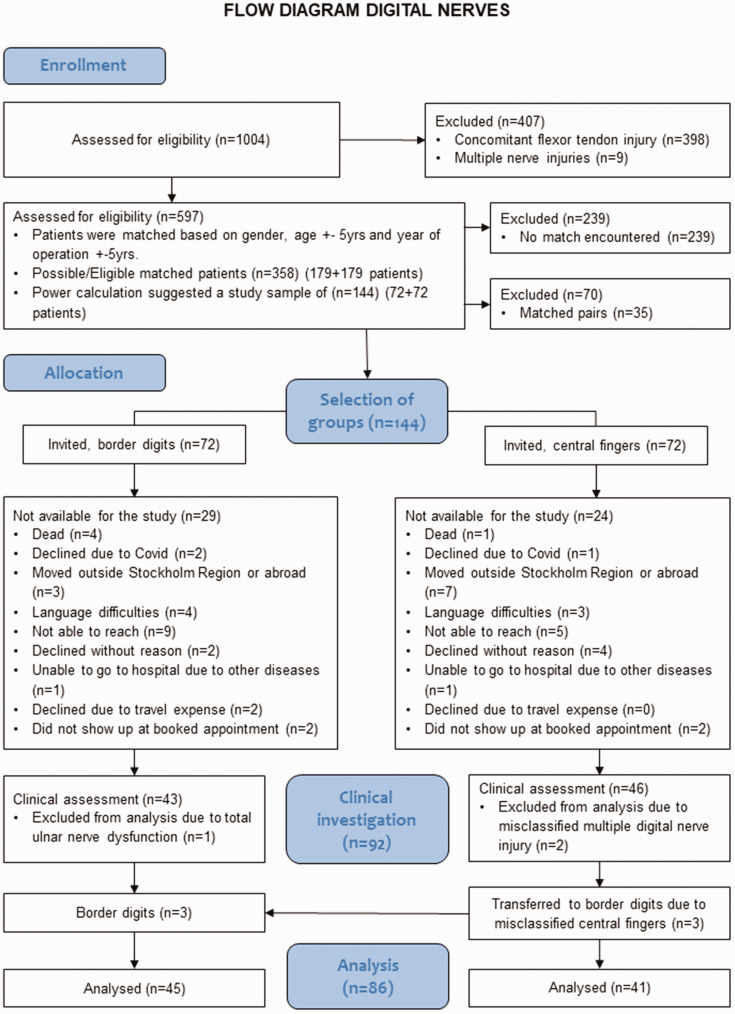
Flowchart of inclusion of patients for evaluation of hand function 3–10 years after digital nerve repair.

**Figure 2.**
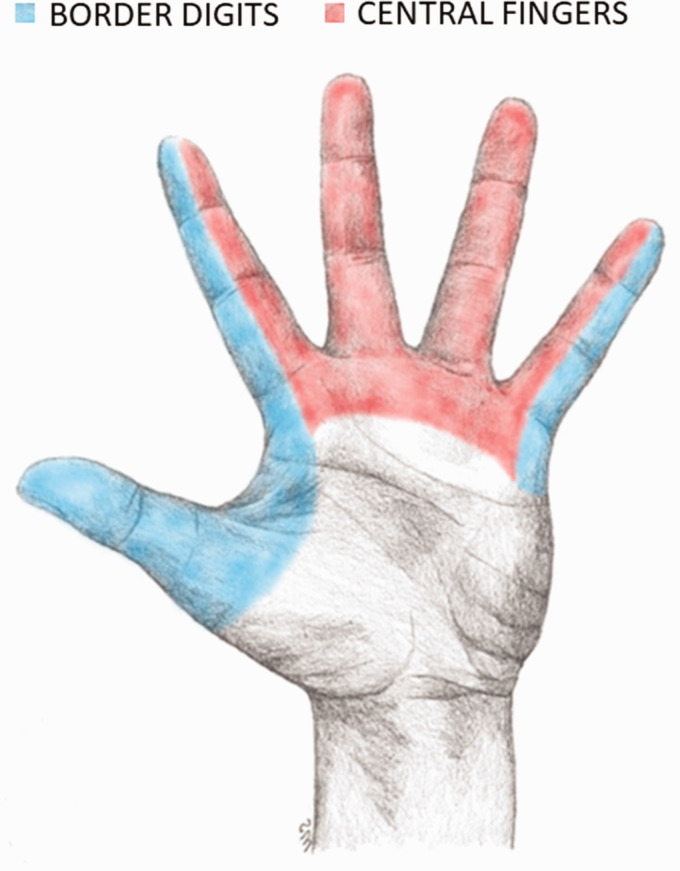
Division between border digits and central fingers in patients with digital nerve injuries.

The inclusion criteria were as follows: diagnostic codes (ICD-10) for a digital nerve injury in the thumb (S64.3) and finger (S64.4); and NOMESCO (Nordic Medico-Statistical Committee) classification of surgical procedures for nerve repair (ACB29) (Swedish version of NOMESCO CoSPV, 1997). Exclusion criteria were as follows: concomitant flexor tendon injury and/or skeletal injury; amputations; multiple digital nerve injuries; and severe soft tissue injuries. Patients unable to communicate in English or Swedish, patients residing outside the Stockholm region and children aged below 18 years were also excluded. A search of the Swedish national quality registry for hand surgery (HAKIR) (Arner, 2016) and the local hospital registry identified 1004 patients with digital nerve injury. Scrutiny of these patients’ medical records identified 407 patents with one or more exclusion criteria, leaving 597 patients with an isolated digital nerve injury (Figure 1). Patients with an injury to a digital nerve in the thumb, radial side of the index finger or ulnar side of the little finger were assigned to the ‘border digits’ group. All other patients were assigned to the ‘central fingers’ group (Figure 2).

The selection of study participants is illustrated in Figure 1. To reduce the risk of bias, matched pairs were created for injury of border digits or central fingers, respectively, based on sex, age ± 5 years and years after surgery ± 5 years. Nine exact matches were found. From the total of 179 possible eligible pairs, after a power calculation, a random sample of 144 patients was selected (72 pairs). Patients were contacted by mail and telephone and invited to participate in a clinical evaluation to investigate hand function in an outpatient clinic setting, free of charge.

### Outcome/data collection

The clinical examinations were conducted by two occupational therapists, previously unknown to the participants, each with over 15 years of experience in assessing hand function. The examinations were performed during a 1-hour outpatient visit at the clinic where the participants were comfortably seated in a quiet room. All tests were conducted on both injured and uninjured hand according to guidelines and gradings of the American Society for Hand Therapists (ASHT) (Casanova, 1992; MacDermid et al., 2015). PROMs included questionnaires completed by the participants during the outpatient visit. Medical records were assessed for age at injury, location and mechanism of injury, surgical technique, smoking habits, time from injury to surgery, and time from injury to sensory relearning in days.

### Hand function

The primary outcome was the Mini Sollerman test (Rosen and Lundborg, 2000). This test comprises tasks 4, 8 and 10 from the Sollerman test (Sollerman and Ejeskar, 1995), which was developed to assess the functional ability to perform hand activities of daily living (Rosén, 1996; Sollerman and Ejeskar, 1995). The Mini Sollerman test assesses three grip types: pulp pinch, where the object is held between the thumb and the index or middle finger; tripod pinch, where the object is surrounded by the thumb, the index and the middle finger; and lateral pinch, where the object is held between the thumb and the radial side of the index finger (Sollerman and Ejeskar, 1995). The total score for the three tasks is calculated for each hand and is in the range of 0–12 (Rosén, 1996). Normal hand function has been reported to be <20 seconds for each task (Singh et al., 2015), which equals a score of 12. Since no previous publication has defined a minimal clinically important difference for the Mini Sollerman test, we determined the clinically importance difference as one after discussion in the study group.

#### Sensory evaluation

Each digital nerve was assessed separately on the radial and ulnar side of the finger pulp. The injured digit was examined only after the completion of evaluations on the corresponding digit of the uninjured hand.

Static 2-point discrimination (S2PD) was used to assess the discriminative touch, i.e. tactile gnosis (Moberg, 1958, 1990). The recommendations of Moberg were used, meaning that the investigator allowed light pressure of a Dellon-Mackinnon Disk-Criminator instrument to be exerted until blanching of the skin occurred (American Society for Surgery of the Hand, 1990; MacDermid et al., 2015). Each distance was applied 10 times in a random order (one/two prongs) and seven answers were needed to be correct before proceeding to a smaller distance (HAKIR, 2018). A value of 6 mm or lower indicates normal sensory function (Casanova, 1992; Lundborg and Rosen, 2004).

The Semmes–Weinstein Monofilament (SWM) test was used to assess the threshold for perception of touch, which reflects re-innervation of cutaneous peripheral receptors. The pocket version with five monofilaments 2.83–6.65 was used (Bell-Krotoski et al., 1995). The test was performed according to the standardized procedure described by Bell-Krotoski et al. (1995). Inability to perceive monofilament 6.65 (300 g) equals no sensation and normal perception of touch is filament 1.65–2.83 (0.008–0.07 g) (Casanova, 1992).

The ability to discriminate warm and cold was investigated by the examiner applying a cold or heated metal probe to the participants’ skin. The ability to identify sharp and dull was assessed by the participant applying a clean safety pin with enough force to create very slight blanching of the skin; the patient was asked to respond if it was perceived as sharp or dull, as previously described by Waylett-Rendall (1988).

### Grip strength and range of motion

Grip strength was assessed using a hydraulic hand dynamometer (Saehan Corp., Changtown, Republic of Korea). Two-point pulp pinch strength was assessed using pinch gauge (Saehan; Saehan Corp., Changtown, Republic of Korea). The results are presented as a percentage of the contralateral side. No correction was performed for injury to the dominant or non-dominant side (Margaliot et al., 2005). Active range of motion was measured with a goniometer. Both grip strength and active range of motion in the finger joints was measured according to the Swedish national manual for measuring motion and strength in the elbow, forearm and hand (Casanova, 1992; HAKIR, 2018).

### Patient-reported outcome measures

Disability was measured with the Swedish version of the short version of the Disabilities of the Arm, Shoulder, and Hand (QuickDASH) questionnaire (Beaton et al., 2005; Gummesson et al., 2006; Hudak et al., 1996). In addition, patients graded their symptoms using the HAKIR patient questionnaire (HQ-8), which includes eight questions graded on a Likert scale in 10-point increments in the range of 0–100, where 0 corresponds to ‘no problem’ and 100 is defined as ‘worst problem imaginable’. The HQ-8 has been investigated for construct validity (Carlsson et al., 2021) and was found to be a valuable complement to the QuickDASH.

Anxiety and depression were evaluated using the Hospital Anxiety and Depression Scale (HADS). HADS is a screening tool for an outpatient clinical setting and consists of two subscales: HADS-anxiety and HADS-depression (Zigmond and Snaith, 1983). Scores are in the range of 0–21 in each subscale, where scores of 8 and above suggest that a disorder may exist.

Participants were also assessed for level of physical activity using the Saltin–Grimby Physical Activity Level Scale (SGPALS), where level of physical activity and exercise are graded on a 4-point scale (physically inactive, light physical activity for at least 4 hours/week, moderate physical activity and training for at least 2–3 hours/week and high-intensity physical training for competitive sports several times/week) (Grimby et al., 2015).

Furthermore, participants filled out the questionnaire Douleur Neuropathique en 4 Questions (DN4), which assesses the probability of neuropathic pain and has been validated for several neuropathic disorders and translated into Swedish (Bouhassira et al., 2005; Hallström and Norrbrink, 2011). Seven self-reported items are included relating to the symptoms and three clinical examinations of the painful area. The total score is 10 and a score of 4 and above suggests the presence of neuropathic pain (Bouhassira et al., 2005).

### Statistical analysis

A sample size calculation was performed before the study. As there were no previous studies showing minimal detectable change or standard deviations using the Mini Sollerman test after digital nerve injury, S2PD was used as a proxy for sample size calculations. With a standard deviation approximated from a previous publication (Mailänder et al., 1989) and assuming a power of 80% and an alpha level of 0.05 of detecting a 20% difference in the proportion of patients with a recovery of 10 mm S2PD or less, we estimated the study population to be 75 cases and 75 controls. However, as the Mini Sollerman test involves more aspects of hand function than only discriminative touch (S2PD), we estimated a sufficient sample to 60 + 60 patients. In addition, we estimated a study dropout of 20%, which resulted in 144 patients who were invited for investigation (Figure 1).

Descriptive statistics were used for patient characteristics. Numbers, proportions and percentages were presented for categorical data. A chi-squared test was used for comparisons of proportions except if the statistical package suggested otherwise. On those occasions, Fisher’s exact test was used. Numerical data were tested for normality using the Shapiro–Wilk test. Median and interquartile range (IQR) were calculated for skewed numerical and ordinal variables. Means and standard deviations (SD) were calculated for the normally distributed continuous variables. All group comparisons for numerical values were carried out using the Mann–Whitney U-test. A conditional logistic regression was performed comparing groups regarding the primary outcome, Mini Sollerman (dichotomized as performed within 20 seconds or not) controlling for dominant hand injury (no/yes), smoking status (no/yes), time from digital nerve injury to surgery <72 hours (yes/no), level of injury (palm/finger) and performance of sensory relearning (no/yes). Correlations were calculated with Spearman’s Rho correlation coefficient for non-parametric data or linear regression when data were normally distributed.

## Results

After extensive attempts to find and contact all 144 potential participants, a total of 86 participants were included (Figure 1). Most injuries affected the non-dominant hand in both the border digit and central finger groups (*n* = 59, 69%). No statistical differences were found in age, smoking, injury characteristics, time to surgery or time to sensory relearning between groups. All participants had been treated with a direct suture of the injured nerve without a nerve graft. Most injuries were sustained at home (*n* = 60, 70%), followed by workplace injuries (*n* = 21, 24%) (Table 1).

**Table 1.** Demographic characteristics of patients with injuries to border digits and central fingers.

Characteristics	Total population (*n* = 86)	Border digits (*n* = 45)	Central fingers (*n* = 41)	*p*-value
Women/Men	40 (47)/46 (53)	21 (47)/24 (53)	19 (46)/22 (54)	0.976^a^
Mean (years)	43 (SD 15)	41 (SD 14)	44 (SD 15)	
Median (years)	45 (26), 19–74	44 (28), 19–64	46 (28), 20–74	0.266^b^
Smoker	26 (37)	13 (34)	13 (41)	0.580^a^
Injury to dominant hand	27 (31)	17 (38)	10 (24)	0.182^a^
Injury mechanism				0.692^a^
Sharp	72 (84)	39 (87)	33 (81)	
Saw	6 (7)	3 (7)	3 (7)	
Crush	1 (1)	0 (0)	1 (2)	
Other	7 (8)	3 (7)	4 (10)	
Level of injury				0.268^c^
Palm	16 (19)	6 (13)	10 (24)	
Digit	70 (81)	39 (87)	31 (76)	
Months from injury to assessment	79 (43), 35–174	82 (41), 36–174	75 (44), 35–118	0.577^b^
Time from injury to surgery (days)				0.398^a^
≤2	44 (51)	21 (47)	23 (56)	
≥3	42 (49)	24 (53)	18 (44)	
Sensory relearning^d^				0.969^a^
Yes	61 (71)	32 (71)	29 (71)	
No	25 (29)	13 (29)	12 (29)	

Data are presented as *n* (%), mean (SD) or median (IQR), range.

a Chi-squared.

b Mann–Whitney U-test.

c Fisher’s exact test.

d Docmented in medical records as received treatment or information regarding sensory relearning.

### Hand function

There was no statistically significant difference (*p* = 0.919) in Mini Sollerman Sum score when comparing participants with border digit injuries with the central finger injuries (Table 2), and a Cox conditional regression analysis confirmed that confounding factors were not significant (Table S1). Only 28 study participants succeeded in performing task 10 (buttoning buttons) within the stipulated 20 seconds (Table S2). When performing a post-hoc analysis of Mini Sollerman Sum score comparing participants with thumb injuries only (*n* = 17) with injuries to all other fingers (*n* = 69), no statistically significant differences in hand function were found (*p* = 0.544).

**Table 2.** Total score and tasks in Mini Sollerman, return of S2PD, discrimination, and physical activity in patients with injuries to border digits and central fingers.

Assessment	Total (*n* = 86)	Border digits (*n* = 45)	Central fingers (*n* = 41)	*p*-value
Mini Sollerman^a^ total score (0–12)	11 (2), 5–12	11 (1.5), 6–12	10 (2.5), 5–12	0.919
Tasks in Mini Sollerman				
Task 4, picking up coins from a purse	4 (0), 2–4	4 (0), 3–4	4 (0), 2–4	0.316
Task 8, putting nuts on bolts	3 (1), 0–4	4 (1), 0–4	3 (1), 0–4	0.308
Task 10, buttoning buttons	3 (1), 0–4	3 (1), 1–4	3 (2), 0–4	0.750
Return of S2PD^b^ according to ASHT classification				
0 points ≥16 mm	11 (13)	4 (9)	7 (16)	0.266
1 point = 11–15 mm	10 (12)	3 (7)	7 (17)	
2 points = 6–10 mm	43 (50)	25 (56)	18 (44)	
3 points = ≤5 mm	22 (26)	13 (29)	9 (22)	
Discrimination^c^				
Intact heat sensation	58 (67)	31 (69)	27 (66)	0.764
Intact cold sensation	68 (79)	36 (80)	32 (78)	0.824
Sharp	57 (66)	30 (67)	27 (66)	0.937
Blunt	67 (78)	36 (80)	31 (75)	0.624
PA according to SGPALS^c^				
Physically inactive	7 (8)	5 (71)	2 (29)	0.212
Some light PA (4 h)	32 (37)	19 (59)	13 (41)	
Regular PA and training	36 (42)	18 (50)	18 (50)	
Regular hard PA for competitive sports	11 (13)	3 (27)	8 (73)	

Data presented as *n* (%) or median (IQR), range.

a Mini Sollerman describes hand function with a Sum score of 12 representing no hand disability, analysed using Mann–Whitney U-test.

b S2PD 6 mm or lower indicates normal values, analysed using Fisher’s exact test.

c Analysed using chi-squared test.

ASHT: American Society for Hand Therapists; PA: physical activity; S2PD: static 2-point discrimination; SGPALS: Saltin–Grimby Physical Activity Level Scale.

### Tactile discrimination

S2PD was measurable in 87% (*n* = 75) of the participants and ranged from 3 mm to inaccurate response even above 15 mm (*n* = 11, 13%), with a median of 7 mm for the whole group. All patients aged 44 years and younger recovered a measurable S2PD (Figure S1). The correlation between age and recovery of S2PD was confirmed in a linear regression (*p* = 0.010, R^2^ = 0.077, y = 5.1 + 0.9*x). There were no statistically significant differences between the groups of border digits or central finger injuries (Table 2). However, 35 (85%) of the participants with border digit injuries recovered S2PD under 10 mm compared to 27 (66%) of the participants with central finger injuries. No significant differences were found between the groups when applying the ASHT classification for S2PD (Table 2).

### Perception of touch

All participants except one in the central fingers group recovered measurable touch perception with SWM. Seventeen (40%) of those with border digit injuries and 19 (44%) with central finger injuries recovered normal perception of touch, i.e. positive identification of SWM monofilament 2.83 (0.07 g). A statistically significant moderate negative correlation was seen between SWM monofilament and S2PD (Spearman’s rho = −0.398, *p* < 0.001). The clinical significance of this finding is that a good performance in SWM monofilament is correlated to a good S2PD result.

### Sensory discrimination

No significant difference was found between the two groups in ability to discriminate cold, warm, sharp and blunt (Table 2).

### Grip strength and finger motion

Grip strength was significantly higher in participants with border digit injuries compared to those with central finger injuries (*p* = 0.027). No significant difference was found in total active range of motion in the finger joints (*p* = 0.515) (Table 3).

**Table 3.** TAM and grip strength, percent of healthy side, in patients with injuries to border digits and central fingers.

	Total (*n* = 85)	Border digits (*n* = 44)	Central fingers (*n* = 41)	*p*-value
TAM	98 (SD 9)99 (11), 76–125	97 (SD 11)99 (14), 76–121	99 (SD 8)99 (8), 87–125	0.515
Grip strength (kg)	93 (SD 21)94 (23), 17–143	98 (SD 19)100 (21), 55–143	87 (SD 21)89 (23), 17–133	**0.027**
Pinch strength	88 (SD 24)89 (25), 22–175	88 (SD 25)88 (26), 38–175	87 (SD 23)89 (23), 22–129	0.940

Data presented as mean (SD) or median (IQR), range. Bold values indicate the statistically significant.

a Analysed using Mann–Whitney U-test.

TAM: total active motion.

### Patient-reported outcome measures

The median QuickDASH score was 5 for the whole study population. No statistical difference was found between participants with border or central digit injuries (*p* = 0.945) (Table 4). HQ-8 scores indicating numbness and cold sensitivity were the worst graded self-reported symptoms in both groups. No significant differences in HQ-8 scores were found between the groups (*p* = 0.590 and *p* = 0.694). The median HADS anxiety score for participants with injuries to border digits was 3 and for central fingers was 5. The median for the HADS depression subscale score was 1 for both groups. No significant differences for either of the subscales were found (Table 4). No significant difference was found between the groups in grading of physical activity according to the Grimby scale (*p* = 0.212) (Table 2).

**Table 4.** PROM results in patients with injuries to border digits and central fingers enrolled in a long-term evaluation of hand function 3–10 years after digital nerve injury and repair.

PROM	Total population			Border digits			Central fingers			*p*-value^a^
QuickDASH	83	13 (SD 18)	5 (18), 0–73	44	12 (SD 16)	6 (18), 0–73	39	15 (SD 21)	5 (23), 0–64	0.945
HQ-8										
Pain on load	86	21 (SD 27)	10 (35), 0–90	45	17 (SD 24)	0 (35), 0–90	41	24 (SD 30)	10 (38), 0–90	0.354
Pain on motion without load	86	11 (SD 20)	0 (10), 0–90	45	8 (SD 17)	0 (10), 0–70	41	14 (SD 23)	0 (20), 0–90	0.521
Pain at rest	86	7 (SD 18)	0 (3), 0–90	45	7 (SD 15)	0 (10), 0–60	41	9 (SD 21)	0 (0), 0–90	0.731
Stiffness	86	16 (SD 23)	10 (20), 0–90	45	14 (SD 21)	10 (20), 0–90	41	18 (SD 25)	10 (30), 0–80	0.730
Weakness	86	16 (SD 24)	0 (30), 0–80	45	12 (SD 21)	0 (15), 0–70	41	20 (SD 27)	10 (30), 0–80	0.105
Numbness/tingling	86	32 (SD 29)	30 (53), 0–90	45	30 (SD 27)	30 (50), 0–90	41	34 (SD 32)	30 (70), 0–90	0.590
Cold sensitivity^b^	86	32 (SD 31)	20 (63), 0–100	45	32 (SD 33)	20 (60), 0–100	41	30 (SD 34)	20 (70), 0–90	0.694
ADL^c^	86	15 (SD 24)	0 (21), 0–90	45	12 (SD 19)	0 (15), 0–70	41	18 (SD 29)	0 (30), 0–90	0.795
HADS										
Anxiety (0–21)	77	5 (SD 4)	3 (5), 0–18	40	4 (SD 4)	3 (4), 0–18	37	5 (SD 4)	5 (6), 0–13	0.181
Depression (0–21)	78	3 (SD 4)	1 (3), 1–15	42	2 (SD 3)	1 (2), 1–13	36	4 (SD 4)	1 (4), 1–15	0.223

Data presented as mean (SD) or median (IQR), range.

a Analysed using Mann–Whitney U-test.

b Discomfort on exposure to cold.

c Ability to perform daily activities.

ADL: activities of daily living; HADS: Hospital Anxiety and Depression Scale; HQ-8: HAKIR patient questionnaire; PROM: patient-reported outcome measure; QuickDASH: short version of the Disabilities of the Arm, Shoulder, and Hand questionnaire.

### Douleur Neuropathique en 4 Questions (DN4)

Of the participants, 19 (44%) in the border digit group and 24 (56%) in the central finger group reported a score of 4 or higher on DN4, indicating presence of neuropathic pain. No statistically significant difference between groups was detected (*p* = 0.131).

## Discussion

In this cohort study, we compared outcomes after digital nerve injuries in border digits and the central fingers 3–10 years after injury and surgical repair. There was no difference in hand function, as measured by the Mini Sollerman test. Grip strength was significantly lower after central finger injuries than after injuries in the thumb, index or little finger, the difference being approximately 10%. It is uncertain if this difference is clinically important.

Isolated digital nerve injuries have been suggested to have little impact on daily life (Dunlop et al., 2019), but our and other studies suggest otherwise (Thorsén et al., 2012). As previously reported, pain and cold intolerance were the most common self-reported symptoms (Collins et al., 1996; Frostadottir et al., 2022; Thorsén et al., 2012). Good sensory recovery after peripheral nerve lesions has been reported to be associated with less neuropathic pain (Magistroni et al., 2020). On the contrary, our study reveals good sensory recovery, but high levels of pain. Further, delayed treatment has been reported to yield worse neuropathic symptoms (de Lange et al., 2022), although not confirmed in our study. A cold climate might have influenced our results regarding neuropathic pain. Although DN4 is recognized for its excellence as a screening tool for peripheral neuropathic pain, further validation may be required specifically for digital nerve injuries (VanDenKerkhof et al., 2018).

Discriminative touch commonly assessed by S2PD (Dunlop et al., 2019) is important for hand function (Novak et al., 1993). However, this instrument has been criticized (Lundborg and Rosen, 2004). Various authors employ diverse definitions of S2PD as a successful outcome, resulting in considerable variation (Dębski et al., 2022; Dunlop et al., 2019; Fakin et al., 2016; Paprottka et al., 2013; Thorsén et al., 2012). SWM is considered more precise than S2PD (Bulut et al., 2016) and a more valid sensory assessment for peripheral nerve injuries (Jerosch-Herold, 2005). However, no single sensory test comprehensively addresses all parameters of sensation and hand function after a digital nerve injury. Incorporating evaluation of cold sensitivity and neuropathic pain in a standard manner, similar to the Rosen score after major nerve injuries (Rosen and Lundborg, 2000), could provide a more comprehensive understanding of results and guide rehabilitation interventions after digital nerve injury.

Research on digital nerve injuries often emphasizes surgical factors over the influence of postoperative rehabilitation. Some authors have questioned the value of repairing single digital nerve injuries distal to the middle phalanx (Pamuk, 2023) or even repairing these nerves at all (Dunlop et al., 2019), due to poor recovery of tactile discriminative touch after surgical repair (Dunlop et al., 2019). However, our study challenges these findings, as all patients aged under 44 years regained a measurable S2PD. Age is an important factor in recovery after nerve injury, favouring younger patients in nerve regeneration and cerebral adaptation (Bulut et al., 2016). Consequently, the necessity for surgical repair of digital nerve injuries in patients aged over 44 years might be less convincing, warranting an informed discussion with them about the low chance of regaining full sensory function. The impact of rehabilitation on sensory recovery is largely unknown and might benefit outcome. In addition, we do not know if measurable 2PD can return even without repair of a digital nerve, e.g. cross over from the adjacent digital nerve. Further investigations are needed.

We compared the outcomes in two groups based on injury location. The Swedish health insurance system assigns patients less insurance compensation for central fingers than for border digits injuries (Insurance Sweden, 2022). However, in our study, grip strength was more affected in patients with central finger injures, which aligns with previous studies on healthy volunteers (Cha et al., 2014). Therefore, we suggest that treatment and rehabilitation should be the same regardless of the affected digital ray.

A strength of this study is the comprehensive incorporation of tests, investigating various parameters of function and disability after digital nerve injury. The large and diverse sample of patients with a long-term follow-up, and the meticulous matching and analysis process using conditional regression, limits confounding factors and improves the generalizability of the results. All examinations followed up-to-date guidelines and were performed by specialized hand therapists. The separate analysis of border digits and central fingers enhances understanding of the impact of the injured digit on functional outcomes.

Limitations concern inclusion and the complexity of assessing hand function. We planned to include 144 participants but were only able to recruit 86, which increases the risk of a type 2 error. Even so, we believe a long-term follow-up of 86 patients can give valuable information. Our matched pairs were selected considering age, sex and time from injury. Another selection could be considered. We used the Mini Sollerman tests as an outcome measure although it has not been validated for digital nerve injuries. However, it is widely accepted for the assessment of major nerve injuries (Rosen and Lundborg, 2003) and is reliable for monitoring changes in hand function (Weng et al., 2010). A strength of this test is the focus on hand function in daily activities, which is more relevant for the patient. Hand function was assessed using QuickDASH. Another questionnaire could have been more appropriate. Another limitation is that we did not perform statistical correction for multiple analysis thus increasing the risk of familywise error rate across the reported statistical analyses. Overall, with this awareness, we encourage other investigators to confirm our findings in future studies.

When comparing patients with digital nerve injury in border digits and central fingers, there was no difference in hand function as measured by the Mini Sollerman test. Sensory recovery is favourable for patients in their 40s or younger, but efforts are needed to minimize neuropathic pain and cold sensitivity.

## Supplemental Material

sj-pdf-1-jhs-10.1177_17531934241286116 - Supplemental material for Long-term subjective and objective outcomes after digital nerve repair: a cohort study Supplemental material, sj-pdf-1-jhs-10.1177_17531934241286116 for Long-term subjective and objective outcomes after digital nerve repair: a cohort study by Linda Evertsson, Anders Björkman, Christina Turesson, Marianne Arner and Cecilia Mellstrand Navarro in Journal of Hand Surgery (European Volume)

## Trial registration

Clinicaltrials.gov: trial registration number 833982.
