# Supplementary figures and images for: Long-term subjective and objective outcomes after digital nerve repair: a cohort study
Source: J Hand Surg Eur Vol. 2024 Oct 13;50(5):649–58. doi: 10.1177/17531934241286116 (PMC12012279; doi:10.1177/17531934241286116)

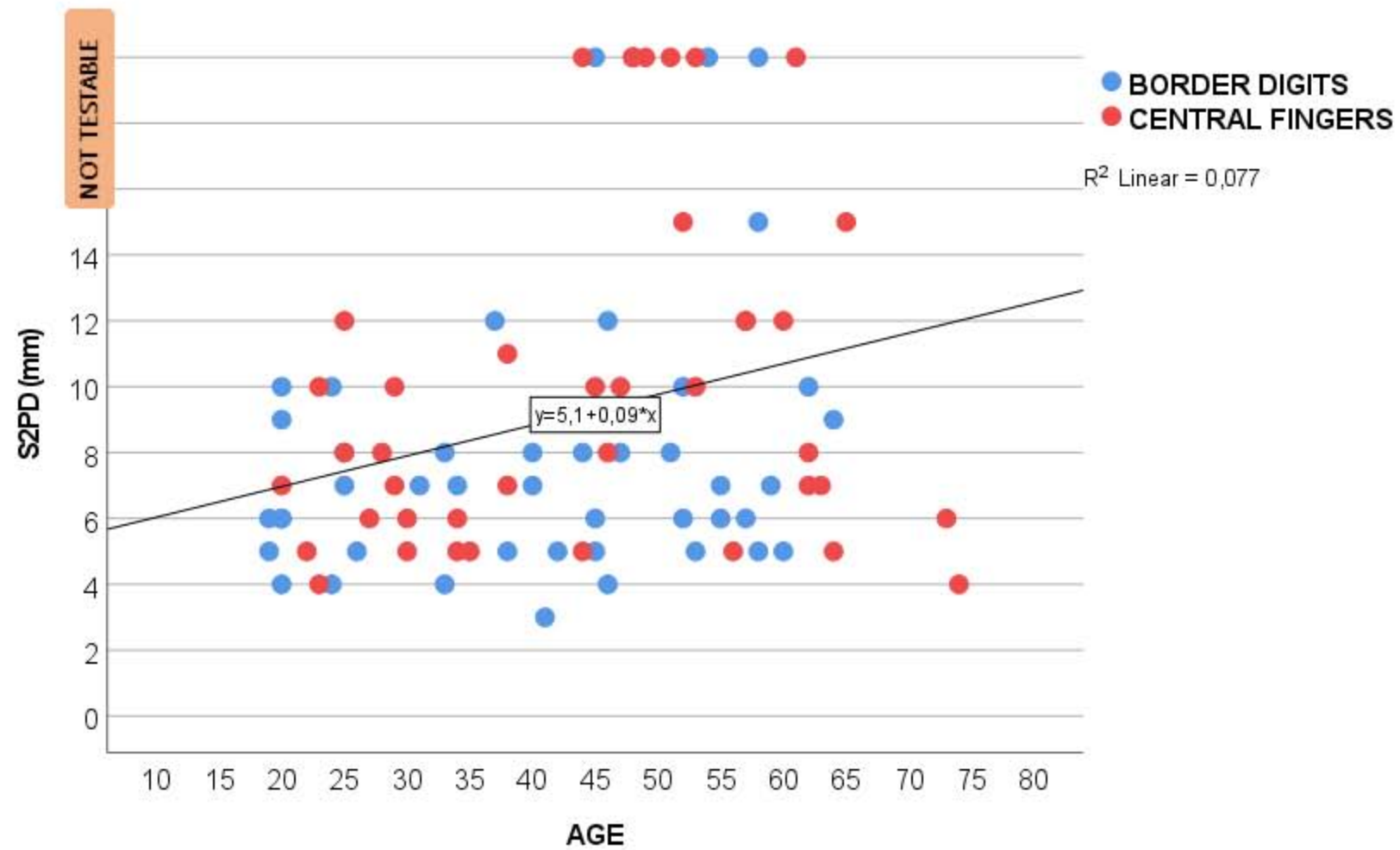

Supplement: sj-pdf-1-jhs-10.1177_17531934241286116 - Supplemental material for Long-term subjective and objective outcomes after digital nerve repair: a cohort study [file sj-pdf-1-jhs-10.1177_17531934241286116.pdf]
